# Syphilis management in pregnancy: a review of guideline recommendations from countries around the world

**DOI:** 10.1080/26410397.2019.1691897

**Published:** 2019-12-30

**Authors:** Thuy Trinh, Alexis F Leal, Maeve B Mello, Melanie M Taylor, Roxanne Barrow, Teodora E Wi, Mary L Kamb

**Affiliations:** a Public Health Analyst, Division of Sexually Transmitted Disease Prevention, Centers for Disease Control and Prevention, Atlanta, GA, USA.; b Project Associate, Department of Communicable Diseases and Environmental Determinants of Health, Pan American Health Organization/World Health Organization, Washington, DC, USA; c Medical Epidemiologist, Regional Adviser for HIV/STI Prevention, Department of Communicable Diseases and Environmental Determinants of Health, Pan American Health Organization/World Health Organization, Washington, DC, USA; d Medical Epidemiologist, Division of Sexually Transmitted Disease Prevention, Centers for Disease Control and Prevention, Atlanta, GA, USA; Medical Officer, Department of Reproductive Health and Research, World Health Organization, Geneva, Switzerland; e Medical Epidemiologist, Division of Sexually Transmitted Disease Prevention, Centers for Disease Control and Prevention, Atlanta, GA, USA; f Medical Officer, Department of Reproductive Health and Research, World Health Organization, Geneva, Switzerland; g Medical Epidemiologist, Division for Parasitic Diseases and Malaria, Center for Global Health, Atlanta, GA, USA

**Keywords:** syphilis in pregnancy, congenital syphilis, mother-to-child transmission of syphilis, guidelines, World Health Organization

## Abstract

Guidelines can help healthcare practitioners manage syphilis in pregnancy and prevent perinatal death or disability. We conducted systematic reviews to locate guidance documents describing management of syphilis in pregnancy, 2003–2017. We compared country and regional guidelines with current World Health Organization (WHO) guidelines. We found 64 guidelines with recommendations on management of syphilis in pregnancy representing 128 of the 195 WHO member countries, including the two WHO guidelines published in 2016 and 2017. Of the 62 guidelines, 16 were for countries in Africa, 21 for the Americas, two for Eastern Mediterranean, six for Europe and 17 for Asia or the Pacific. Fifty-seven (92%) guidelines recommended universal syphilis screening in pregnancy, of which 46 (81%) recommended testing at the first antenatal care visit. Also, 46 (81%) recommended repeat testing including 21 guidelines recommended this during the third pregnancy trimester and/or at delivery. Fifty-nine (95%) guidelines recommended benzathine penicillin G (BPG) as the first-line therapy for syphilis in pregnancy, consistent with WHO guidelines. Alternative regimens to BPG were listed in 42 (68%) guidelines, primarily from Africa and Asia; only 20 specified that non-penicillin regimens are not proven-effective in treating the fetus. We identified guidance recommending use of injectable penicillin in exposed infants for 112 countries. Most guidelines recommended universal syphilis testing for pregnant women, repeat testing for high-risk women and treatment of infected women with BPG; but several did not. Updating guidance on syphilis testing and treatment in pregnancy to reflect global norms could prevent congenital syphilis and save newborn lives.

## Résumé

Les principes directeurs peuvent aider les praticiens de santé à prendre en charge la syphilis pendant la grossesse et prévenir la mortalité ou le handicap périnatal associé. Nous avons mené une étude systématique et structurée pour répertorier les guides décrivant la prise en charge de la syphilis de 2003 à 2007. Nous avons comparé les directives nationales et régionales avec les principes directeurs actuels de l’Organisation mondiale de la santé (OMS). Nous avons recensé 64 directives avec des recommandations sur la prise en charge de la syphilis pendant la grossesse représentant 128 (66%) des 195 États membres de l’OMS, notamment deux principes directeurs de l’OMS publiés en 2016 et 2017. Des 62 directives nationales et régionales, 16 concernaient des pays en Afrique, 21 aux Amériques, deux en Méditerranée orientale, six en Europe alors que 17 se rapportaient à des pays d’Asie et du Pacifique. Cinquante-sept (92%) principes directeurs préconisaient un dépistage universel de la syphilis pendant la grossesse, dont 46 (81%) conseillaient de pratiquer le test lors de la première visite prénatale. De même, 46 (81%) recommandaient de répéter le test (dont 21 des 46 directives pendant le troisième trimestre de la grossesse et/ou au moment de l’accouchement). Cinquante-neuf (95%) principes directeurs préconisaient la benzathine-pénicilline G (BPG) comme traitement de première intention pour la syphilis pendant la grossesse, conformément aux directives de l’OMS. Des traitements autres que la BPG figuraient dans 42 (68%) principes directeurs, principalement d’Afrique et d’Asie; seulement 20 (48%) principes directeurs précisaient que l’efficacité des régimes médicamenteux sans pénicilline n’est pas prouvée pour traiter le fœtus. Nous avons identifié des directives recommandant l’emploi de pénicilline injectable chez les nourrissons exposés dans 112 (87%) pays. Lorsque les principes directeurs existent, la plupart recommandent un dépistage universel de la syphilis chez les femmes enceintes, une répétition du test pour les femmes à risque élevé et le traitement des femmes infectées avec la BPG; mais plusieurs ne le font pas. La mise à jour des guides sur le dépistage et le traitement de la syphilis pendant la grossesse pour tenir compte des normes mondiales pourrait prévenir la syphilis congénitale et sauver la vie de nouveau-nés.

## Resumen

Las directrices pueden ayudar a los profesionales de la salud a manejar la sífilis durante el embarazo y evitar la muerte o discapacidad perinatal asociada con esta enfermedad. En 2003–2017, realizamos una revisión sistemática estructurada para localizar documentos de orientación que describen el manejo de la sífilis durante el embarazo. Comparamos las directrices nacionales y regionales con las directrices vigentes de la Organización Mundial de la Salud (OMS). Encontramos 64 directrices con recomendaciones sobre el manejo de sífilis durante el embarazo, que representan 128 (66%) de los 195 Estados miembros de la OMS, entre ellas las dos nuevas guías de la OMS publicadas en 2016 y 2017. De las 62 directrices nacionales y regionales,16 eran para países en África, 21 para las Américas, dos para el Mediterráneo oriental, seis para Europa y 17 para países en Asia o el Pacífico. El 92% (57)de las directrices recomendaron tamizaje universal de sífilis durante el embarazo, de las cuales el 81% (46) recomendaron tamizaje en la primera consulta de atención prenatal. Además, el 81% (46) recomendaron pruebas repetidas (21 de las 46 directrices durante el tercer trimestre del embarazo y/o después del parto). El 95% (59) de las directrices recomendaron penicilina G benzatina (PGB) como terapia de primera línea para la sífilis durante el embarazo, en conformidad con las directrices de la OMS. En 42 directrices (68%), se mencionaron regímenes alternativos a PGB, principalmente de África y Asia; solo 20 (48%) especificaron que los regímenes sin penicilina no han sido comprobados como eficaces para el tratamiento del feto. Identificamos orientación que recomienda el uso de penicilina inyectable en bebés expuestos para 112 países (87%). Donde existen directrices, la mayoría recomienda tamizaje universal de sífilis en mujeres embarazadas, pruebas repetidas en mujeres de alto riesgo y tratamiento de mujeres infectadas con PGB; pero varias no recomiendan estas medidas. La actualización de la orientación sobre las pruebas de sífilis y el tratamiento durante el embarazo para reflejar las normas mundiales podría prevenir la sífilis congénita y salvar la vida de recién nacidos.

## Introduction

In 2016, the World Health Organization (WHO) estimated there were 6.3 million new cases of syphilis, representing a prevalence of 0.5% in both men and women,^1^ and 0.69% among pregnant women.^2^

Syphilis is transmitted via sexual exposure or from mother to child during pregnancy. When left untreated, maternal syphilis is estimated to result in adverse birth outcomes (ABOs) in 50−80% of affected pregnancies, depending upon the stage of syphilis in the woman.^3–5^ ABOs associated with syphilis are often severe, with stillbirth (most commonly) or neonatal death accounting for more than half of poor outcomes. Prematurity, low birth weight and congenitally infected infants are also common if syphilis in pregnancy is untreated or treated late.^5,6^ Coupled with early detection of cases through testing, prompt treatment with parenteral penicillin cures maternal and fetal infections and can prevent these adverse pregnancy outcomes. Economic analyses have demonstrated that syphilis screening and treatment in pregnancy are among the most highly cost-effective public health interventions available, and can be cost-saving in some countries.^7^ Since syphilis infections in adults and infants are often asymptomatic or unrecognised, maternal screening is a critical intervention. For this reason, WHO includes as part of the congenital syphilis case definition, exposure based on maternal syphilis screening and adequate treatment with penicillin.^8^

Syphilis screening and treatment for pregnant women and their sex partner(s)^9^ can help practitioners manage syphilis effectively and prevent adverse health outcomes in the mother, her sex partner(s) and infant(s), and reduce transmission. Previous WHO guidelines for management of sexually transmitted infections (STIs) from 2003 were focused on management based on symptoms due to the lack of or limited diagnostics available in many low- and middle-income countries.^10^ However, because STIs are often asymptomatic for both men and women, management approaches based only on symptoms miss a large proportion of new and prevalent cases. Such “syndromic approaches” can also hamper effective partner management strategies, as there is no definitive STI diagnosis without a laboratory test. In developing their current evidenced-based recommendations, the Guideline Development Group of WHO used the Grading of Recommendation Assessment Development and Evaluation (GRADE) methodology. Using GRADE, WHO published updated guidelines on management of syphilis,^3^ gonorrhea,^11^ Chlamydia^12^ and genital herpes^13^ in 2016. Additionally, WHO provided the first specific guidelines on *Syphilis Screening and Treatment for Pregnant Women*, published in 2017, in which syphilis testing was recommended for all pregnant women at the first antenatal care visit, and benzathine penicillin G (BPG) as the first-line treatment. Recommendations were also provided on treatment and follow up of exposed infants.^14^ WHO encouraged countries to update their national guidelines on syphilis management using the new 2016 and 2017 WHO recommendations. Here, we review national STI guidelines from 2003 to 2017 for countries around the world to compare them with the current WHO guidelines on syphilis screening, treatment and management among pregnant women. This review is important to ensure that national guidelines have adopted the new recommendations promoting early detection and treatment of syphilis in pregnancy, thus preventing the 61,000 neonatal deaths, 41,000 premature or low birth weight infants, 143,000 early fetal deaths and stillbirths and 109,000 infants with clinical disease estimated to occur annually due to congenital syphilis.^2^

## Methods

### Search strategy and inclusion criteria

We performed a systematic review that followed Preferred Reporting Items for Systematic Reviews and Meta-Analyses (PRISMA) requirements.^15^ We sought to identify national guidelines published in multiple languages since 2003 (the publication date of the most recent previous WHO guidance on STI management) describing the management of syphilis in pregnancy. To identify national guidelines, we conducted internet searches and structured, systematic reviews of PubMed, Geneva Foundation for Medical Education and Research, Clinton Health Access Initiative, and Google Search to locate documents describing management of syphilis for pregnant women. We used the following search terms: “National Guidelines for the Management of Sexually Transmitted Infections”, “National Guidelines on Sexually Transmitted Infection Case Management”, “Sexually Transmitted Diseases treatment guidelines”, “Sexually Transmitted Diseases management guidelines”, “Sexually Transmitted Disease treatment and management guidelines”, “STD treatment guidelines”, “STD treatment and management guidelines”, “Sexually Transmitted Infections treatment guidelines”, “STI management”, “STI management and treatment guidelines”, “Reproductive tract infections (RTI) treatment guidelines”, “Reproductive tract infections management guidelines”, “Reproductive tract infections treatment and management guidelines”, “RTI treatment and management guidelines”, “STI and RTI treatment and management guidelines”, and “National Guidelines on elimination of congenital syphilis”. Additionally, we searched WHO websites and contacted WHO regional advisors and consultants, and local technical staff to identify guidance documents used in countries in their regions, regardless of language, in the above topics, as well as Maternal and Child Health, Prevention of Mother-to-Child Transmission, and Reproductive Health guidelines, technical updates, or other related guidance documents. We identified native speakers to assist in translating most documents, including all Spanish, Portuguese and French language documents, and in a few instances used Google Translate. For countries with more than one document identified, we used the most recently published document that described management of syphilis in pregnancy. Also, for countries with more than one recent guidance document (e.g. separate guidance documents for STI treatment and management of syphilis for pregnant women), all documents were considered to be a single “package” of guidelines. We focused our review on countries and did not include territories unless their guidance documents were substantially different from their administrating nations. For example, we did not include guidelines from territories (e.g. Puerto Rico or U.S. Virgin Islands) that followed their national or regional guidance.

### Review process

Prior to reviewing the guidelines, we collaborated with regional experts and co-authors to generate standardised review criteria that included 53 discrete variables that were assessed for each document using an Excel spreadsheet. The variables fell under the general topics of specific screening recommendations (e.g. for all women, when during pregnancy, repeat screening, at delivery), testing conducted (algorithm, type of test), and treatment/dosing recommended (e.g. only BPG, other antibiotic regimens) by stage for pregnant women, infants and partners. We then reviewed country guidelines using these criteria and variables, recording relevant data for each as appropriate using Microsoft Excel 2016. To ensure consistency, we worked with WHO regional experts and co-authors whenever clarification or refinement in variable definitions was required. For example, for the variable “syphilis described as a ‘high-risk pregnancy’”, we determined that a “yes” answer could be used if an array of adverse pregnancy outcomes was described, even if the words “high-risk pregnancy” were not included. Also, for the variable “partner therapy mentioned,” we marked “yes” if partner management was described under STI prevention and treatment section; and we marked “partner treatment not specified” if partner treatment was not clearly recommended in the national guidelines. We used definitions provided in the specific guideline for “early” and “late latent” syphilis (i.e. used a cut-off of one year in North American and European, Centers for Disease Control and Prevention (CDC) guidelines, and two years for WHO and other guidelines). However, we used the WHO definition for congenital syphilis, (i.e. live or stillborn infant, or fetal loss >20 weeks gestation or 500 g, born to a mother who was not treated with at least one dose of 2.4 million units (MU) intramuscularly (IM) penicillin at least 30 days prior to delivery, or infant or child <2 years with clinical, laboratory or x-ray evidence of syphilis infection). We compared the country and regional guidance documents to the 2016 WHO guidelines for the treatment of *Treponema pallidum* (syphilis) and the 2017 guidelines for syphilis screening and treatment for pregnant women.

### Statistical analysis

We reported on countries geographically according to the six WHO regions as being part of the African Region (AFR), Americas Region (AMR), European Region (EUR), Eastern Mediterranean Region (EMR), South East Asian Region (SEAR) or West Pacific Region (WPR).^16^ When specific country guidelines were not available, we attempted to identify international regional guidelines that may have reflected management of syphilis for those countries. Additionally, we used WHO country-reported data to compare syphilis screening and diagnostic algorithms recommended for pregnant women in countries according to maternal prevalence in each country, using the most recently reported data by countries through 2015. For this subanalysis, we used seroprevalence cutoffs of <1%, and ≥1% to describe lower and higher country prevalence, respectively.^8^ We also used these seroprevalence cutoffs to examine treatment of exposed infants using WHO country reported data on treatment. Statistical analyses were done using Microsoft Excel 2013 (Microsoft, Redmond, WA, USA), and comparisons were made using SAS 9.3 (Cary, NC, USA).

## Results

**Figure 1.**
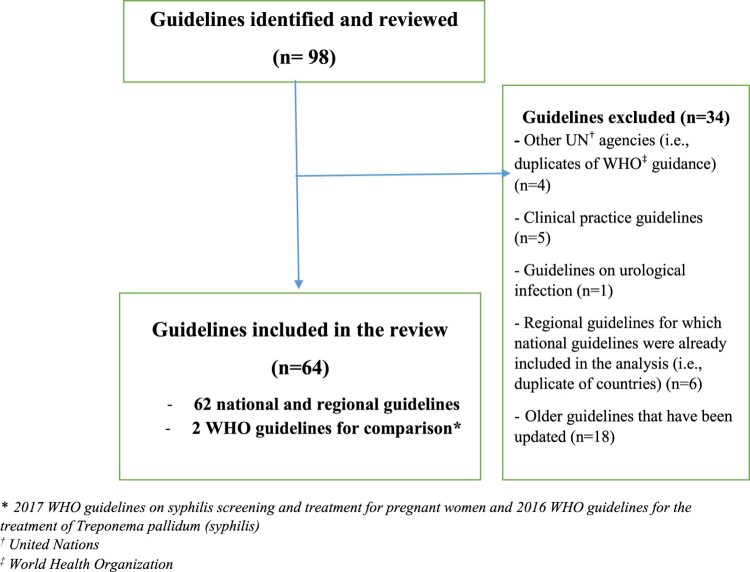
Identification of national guidelines describing screening, treatment and management of syphilis in pregnancy

We identified and reviewed 98 STI treatment and other national guidelines on screening, treatment and management of syphilis in pregnancy published during 2003–2017. We excluded 34 that were duplicate guidelines (Figure 1). We used two WHO guidelines as comparison guidelines. The 62 guidelines, representing 128 countries, were evaluated (Figure 1). We found no evidence of national or regional guidance addressing management of syphilis in pregnancy for the remaining 67 WHO member states, of which most (46, or 69%) were low- or lower-middle-income countries.

### WHO guidelines

The WHO guideline on *Syphilis Screening and Treatment for Pregnant Women* published in 2017 recommends screening all pregnant women for syphilis during the first antenatal care visit. It further recommends that in settings with low coverage of syphilis screening and/or treatment, or in settings with limited laboratory capacity, a single on-site test such as rapid diagnostic test (RDT) or on-site rapid plasma reagin (RPR) should be used, citing the urgency of ensuring prompt treatment in pregnancy to avoid congenital syphilis.

In 2016, WHO issued a guideline for the treatment of *T. pallidum*. This document recommends that pregnant women with early syphilis (primary, secondary or early latent syphilis) be treated with BPG 2.4 million units (MU) once intramuscularly (IM) as a single dose or, as a back-up when BPG was unavailable, procaine penicillin 1.2 MU IM once daily for 10 days. The guideline recommends treatment among pregnant women with late latent syphilis or infection of unknown duration with BPG 2.4 MU IM once weekly for three consecutive weeks. It notes that “when benzathine or procaine penicillin cannot be used (e.g. due to penicillin allergy where penicillin desensitisation is not possible) or are not available (due to stock-outs)”, erythromycin 500 mg orally four times daily for 14 days, or ceftriaxone 1 g IM once daily for 10–14 days, or azithromycin 2 g orally can be used, with caution. This WHO guideline specifically states that erythromycin and azithromycin can treat pregnant women, but do not cross the placental barrier completely and as a result do not reliably treat the fetus; and thus, any infant born to a syphilitic woman who was not treated with BPG must be considered to have congenital syphilis and managed accordingly. The document further recommended against use of doxycycline during pregnancy for safety reasons. The guideline strongly recommends that national programmes proactively forecast penicillin needs carefully to avoid stock-outs “because syphilis during pregnancy can lead to severe adverse complications” in an exposed fetus or newborn.

For infants with congenital syphilis, the document recommends treatment with aqueous benzyl penicillin 100,000–150,000 U/kg/day intravenously for 10–15 days, or procaine penicillin 50,000 U/kg/day single dose IM for 10–15 days. If an infant is considered to be at very low risk of congenital syphilis (i.e. exposed to syphilis but clinically normal and whose mother’s syphilis infection had been adequately treated with no signs of reinfection or had very low maternal RPR titers), BPG 50,000 U/kg/day single dose IM is an option.

### Description of country guidelines

Of the 62 national and regional guidelines evaluated, 21 (34%) were from AMR, 16 (26%) from AFR, 11 (17%) from WPR, six (10%) from EUR, six (10%) from SEAR and two (3%) from EMR. The 62 guidelines represented 128 countries, as 48 countries in EUR used the European guidelines,^17^ seven countries from AFR used the Southern African Development Community guidelines,^18^ six countries from AMR used the Organization of Eastern Caribbean States (OECS) guidelines,^19^ and 10 Pacific Island countries in WPR used the 2016 WHO guidelines. Three countries had more than one guidance document (e.g. separate guidance for treatment of syphilis and management of syphilis in pregnancy) that were considered as a single “package of guidelines” for the country (Table 1).

**Table 1.** Regional and national guidelines included in the review, by World Health Organization (WHO) region (n = 62 guidelines representing 128 countries)

**African Region** (16 guidelines, 22 countries)	**Americas Region** (21 guidelines, 26 countries)	**Eastern Mediterranean Region** (2 guidelines, 2 countries)	**European Region**(6 guidelines, 53 countries)	**South East Asia Region** (6 guidelines, 5 countries)	**Western Pacific Region** (11 guidelines, 20 countries)
Ethiopia Ghana Kenya* Liberia Malawi Mozambique Namibia Nigeria Rwanda South Africa Swaziland Tanzania Uganda Zambia Zimbabwe Southern African Development Community^†^(used by other 7 member countries)	Argentina Bolivia Brazil Canada Chile Colombia Cuba Dominican Republic Ecuador El Salvador Guatemala Honduras Jamaica Mexico Nicaragua Paraguay Peru United States and U.S. territories (CDC) Uruguay Venezuela OECS HIV/STI Guidelines 2017^‡^(used by 6 member countries)	Egypt Pakistan	Estonia Greece Romania Montenegro United Kingdom European Guidelines^§^(used by other 48 member countries)	India Indonesia Nepal Sri Lanka* Thailand SEARO regional guidelines	Australia Cambodia Hong Kong* Japan Malaysia New Zealand Palau Philippines Singapore Vietnam Pacific Community Guidelines (10 Pacific island countries used WHO 2016 guidelines)^¶^

*Have more than one guideline covering management of syphilis in pregnancy: Hong Kong, Sri Lanka and Kenya.

^†^Angola, Botswana, Democratic Republic of Congo, Lesotho, Madagascar, Mauritius, Seychelles.

^‡^Antigua and Barbuda, Dominica, Grenada, Saint Kitts and Nevis, Saint Lucia, Saint Vincent and the Grenadines.

^§^Albania, Andorra, Armenia, Austria, Azerbaijan, Belarus, Belgium, Bosnia and Herzegovina, Bulgaria, Croatia, Cyprus, Czech Republic, Denmark, Kyrgyzstan, Finland, France, Georgia, Germany, Hungary, Iceland, Ireland, Israel, Italy, Kazakhstan, Latvia, Lithuania, Luxembourg, Malta, Monaco, Netherlands, Norway, Poland, Portugal, Republic of Moldova, Russian Federation, San Marino, Serbia, Slovakia, Spain, Sweden, Switzerland, Tajikistan, North Macedonia, Turkey, Turkmenistan, Ukraine, Uzbekistan.

^¶^Kiribati, Cook Islands, Marshall Islands, Micronesia, Nauru, Niue, Samoa, Tonga, Tuvalu, Vanuatu.

Of the 62 national or regional guidelines, 38 (62%) were aimed at STI management, 17 (27%) were guidelines on perinatal care, two (3%) were aimed at reproductive tract infection management and five (8%) were general guidelines such as syndromic case management guidelines, standard treatment guidelines for diagnosis and treatment of infection or other common conditions. Most guidelines (73%) included a specific section that detailed separate, special screening, treatment and management of syphilis during pregnancy. Seventeen documents (27%) were focused primarily on the management of clinical syphilis in adults and mentioned management during pregnancy only in passing (no specific section).

### Comparison of country guidelines to WHO guidelines

#### Screening

Of the 62 guidelines, universal syphilis screening in pregnancy was recommended by 57 (92%). Four national and one regional guidelines in WPR did not recommend universal syphilis screening during pregnancy. Of the 57 documents recommending universal screening, 46 (81%) recommended syphilis screening early in pregnancy, either in the first trimester or at the first antenatal care (ANC) visit, consistent with WHO recommendations. Additionally, among 46 guidance documents recommending repeat screening during pregnancy, 21 (46%) did so in the 3rd trimester and/or at delivery, beyond current WHO recommendations. Of the 62, 47 (76%) documents described at least one diagnostic algorithm for syphilis screening, consistent with WHO recommendations. Of those, 38 (81%) recommended a traditional algorithm (i.e. non-treponemal test confirmed by treponemal test), 31 (66%) recommended a reverse algorithm (i.e. treponemal test first then confirmed by one or more non-treponemal tests) and 38 (81%) recommended both algorithms. Among the 31 guidelines that recommended a reverse algorithm, 20 (65%) suggested RDT as the first test, which is promoted by WHO for settings where serologic testing, or results, are unavailable (Table 2).^20,21^

**Table 2.** Syphilis screening, treatment and management recommendations in pregnant women (n = 62 guidelines)

Recommendation	n (%)	Consistent with WHO recommendations
**Screening** Universal First screening early in pregnancy Repeat screening during 3rd trimester and/or at delivery	57/62(92) 46/57(81) 21/46* (46)	Yes Yes Yes
**Laboratory diagnosis** Algorithm recommended (at least one) Traditional algorithm^†^ Reverse algorithm^‡^	47/62 (76) 38/47^§^ (81) 31/47^§^ (66)	Yes Yes Yes
**Treatment recommendations** Recommended injectable penicillin as first-line therapy Recommended BPG 2.4 MU IM for early syphilis and 7.2 MU for late syphilis Recommended BPG 7.2 MU IM in 3 weekly doses, regardless of stage Recommended.BPG 2.4 MU IM as a single doses regardless of stage Allowed non-penicillin regimens to be included as alternative treatment	59/62 (95) 45/59 (76) 8/59^¶^ (14) 6/59^¶^ (10) 42 (68)	Yes Yes No No Yes
**Desensitisation recommended for penicillin-allergic women**	31 (50%)	Yes

*Of the guidance documents recommending repeat screening during pregnancy.

^†^Non-treponemal test done first and confirmed with a treponemal test.

^‡^Treponemal test done first and confirmed with one or more non-treponemal tests.

^§^Documents described at least one diagnostic algorithm for syphilis screening.

^¶^Among guidelines recommended treatment with injectable penicillin.

#### Treatment and management of mothers

Of the 62 guidelines, 34 (55%) noted that maternal syphilis infection resulted in a high-risk pregnancy. Fifty-nine (95%) guidelines recommended treatment with injectable penicillin as the first-line treatment; however, six of these (10%) recommended only single-dose management regardless of syphilis stage (less than WHO-recommended management), and another eight (14%) recommended using three doses regardless of stage or unknown duration (more than WHO-recommended management). These 14 documents were focused primarily on management syndromes of genital ulcer disease (i.e. including management of syphilis in the absence of laboratory testing).

Forty-two (68%) guidelines allowed alternative (non-parenteral penicillin) regimens for treatment of pregnant women with syphilis. Alternative regimens included oral erythromycin, IM ceftriaxone, oral azithromycin or oral amoxicillin. Only 20 (48%) of these documents specifically noted that the fetus was not treated if an alternative regimen was used. Thirty-one guidelines (50%) recommended desensitisation for women allergic to penicillin (including 12 countries that allowed alternative treatment, but preferred penicillin regimens with desensitisation where feasible) (Table 2). Nineteen guidelines (32%) recommended treatment only with penicillin, even in allergic women who would thus require desensitisation.

Overall, relatively few (n = 12) guidance documents described global penicillin shortages or the need to forecast to ensure sufficient penicillin was available, as recommended in WHO guidance. Guidelines from one country in SEAR and one country in AFR specifically described forecasting of BPG. Another country in AFR had guidelines that mentioned shortages of drugs needed to treat STIs without specifically describing the importance of forecasting penicillin needs. One country in EUR and one country in WPR had guidelines cautioning that stock-outs of penicillin should be avoided. A guideline from one country in SEAR recommended that BPG be made available at maternity wards. Over half (n = 7) of the 12 guidelines noting the importance of ensuring adequate medications and/or supplies for management of syphilis or STIs in general were from AMR. In this region, six of the seven country guidelines noted the responsibilities and procedures needed to ensure adequate generalised STI supplies and medications and one country guideline called attention to structural factors that may affect the availability of penicillin in institutions providing services to syphilis patients. Of the six guidelines that noted responsibilities and procedures, one country’s guideline called for ensuring adequate medical and surgical supplies needed for management and treatment of pregnant syphilis- and HIV-infected women and exposed fetuses while another country’s guideline called for sub-ministries and laboratories to prioritise forecasting general medication needs to meet demand.

#### Partner management

Overall, 59 (95%) of the 62 guidelines mentioned the importance of partner treatment, which is not specifically addressed in the new WHO guidelines. Of these, 55 (93%) guidelines provided information on how to manage partners, of which 29 (53%) recommended using the same syphilis treatment for partners as was used for pregnant women, and 32 (58%) recommended penicillin as the first-line therapy for sex partners. Nine guidelines (16%) mentioned expedited partner management as a possible STI prevention strategy; this would suggest that they were promoting oral agents for partners rather than injectable penicillin.

#### Syphilis screening recommendations among higher and lower prevalence countries

Maternal syphilis seroprevalence was reported to WHO by 115 (90%) of the 128 represented countries. Of these, 73 (63%) countries reported that maternal syphilis prevalence was less than 1% and 42 countries reported prevalence was at least 1% or higher. Higher and lower seroprevalence countries did not differ on recommendation of universal screening among pregnant women. However, countries with maternal syphilis prevalence less than 1% were more likely than higher seroprevalence countries to recommend the first syphilis screening during the first trimester of pregnancy (69% vs. 29%, *p* < 0.05). Among the 102 countries recommending repeat screening, 40% of country guidelines recommended repeat screening later in pregnancy (second and third trimester), and 92% of countries recommended repeat screening during delivery. Countries with maternal syphilis prevalence at least 1% or higher were more likely than lower prevalence countries to recommend repeat screening for all pregnant women (48% vs. 27%, *p* < 0.05). Repeat screening was recommended more commonly only for women at high risk of infection in countries with maternal syphilis prevalence less than 1% than in countries with higher prevalence (62% vs. 16%. *p* < 0.05) (Table 3).

**Table 3.** Syphilis screening recommendations in pregnant women, by level of maternal syphilis seroprevalence in country (n = 115 countries*)

	Maternal syphilis prevalence <1% (n = 73 countries)	Maternal syphilis prevalence ≥1% (n = 42 countries)	*p*-value
**Universal screening**			
Recommended	71 (97%)	40 (95%)	0.57
**Time of the first screening**			
At the first trimester	50 (69%)	12 (29%)	<0.01
At the first ANC visit	17 (24%)	23 (55%)	
Not reported	6 (7%)	7 (16%)	
**Repeat screening during pregnancy**			
Yes, universal	20 (27%)	20 (48%)	<0.01
Yes, only high risk	45 (62%)	7 (16%)	
Not reported	8 (11%)	15 (36%)	
**Screening during delivery**			
Yes, always	11(15%)	11 (26%)	<0.01
Yes, if no evidence of prior screening	3 (4%)	2 (5%)	
Yes, if at risk	45 (62%)	10 (24%)	
Not reported	14 (19%)	19 (45%)	

*13 countries had no available maternal syphilis seroprevalence data.

#### Comparison of treatment recommendations by region

Consistent with the WHO recommendations, 78% of countries in AMR, 90% in WPR, 95% in AFR, 98% EUR and 100% in SEAR recommended BPG 2.4 MU IM in a single dose as the first-line therapy to treat early syphilis during pregnancy. Additionally, 90% or more countries in WPR, EUR and SEAR recommended 7.2 MU IM in three weekly doses of 2.4 MU each to treat late latent syphilis consistent with WHO recommendations. However, there are two countries in AFR and six countries in AMR that recommended three doses of BPG 2.4 MU IM as the first-line therapy regardless of syphilis stage (beyond WHO recommendations). Additionally, non-penicillin regimens were recommended as alternatives to penicillin for treatment of syphilis during pregnancy in guidelines of 8% of countries in EUR, 31% of countries in AMR, 85% of countries in WPR, 91% in AFRO and 100% in SEAR and EMRO. Eighteen per cent of guidelines for AFR countries and 20% of SEAR countries recommended desensitisation for penicillin-allergic women, compared to 73% or more countries in AMR, WPR and EUR (Table 4).

**Table 4.** Treatment recommendations for pregnant women, by World Health Organization (WHO) region (n = 128 countries)

	African Region (n = 22)	Americas Region (n = 26)	Eastern Mediterranean Region (n = 2)	European Region (n = 53)	South East Asian Region (n = 5)	Western Pacific Region (n = 20)
Treatment recommended according to WHO guidelines	n (%)	n (%)	n (%)	n (%)	n (%)	n (%)
Early syphilis* BPG^†^ 2.4 MUIM single dose	20 (95%)	20 (78%)	2 (100%)	52 (98%)	5 (100%)	18 (90%)
Late latent syphilis BPG 7.2 MU IM in three weekly doses	16 (73%)	20 (77%)	1 (50%)	52 (98%)	5 (100%)	18 (90%)
BPG 7.2 MU IM in three weekly doses regardless of stage	2 (9%)	6 (23%)	0	0	0	0
Non-penicillin regimens were included as alternatives	20 (91%)	8 (31%)	2 (100%)	4 (8%)	5 (100%)	17 (85%)
Desensitisation recommended for penicillin allergic women	4 (18%)	19 (73%)	0 (0%)	53 (100%)	1 (20%)	16 (80%)

*Regarding early syphilis (primary, secondary and early latent syphilis).

†BPG = benzathine penicillin G.

Several countries’ guidelines recommended management of syphilis during pregnancy that was clearly inconsistent with WHO guidelines. Four countries in AFR and one country in WPR recommended treatment of BPG 2.4 MUIM (single dose only); four of these were focusing solely on genital ulcer disease while one mentioned treatment of pregnant women seropositive for syphilis without mention of stage; and none of these mentioned treatment of late latent syphilis or syphilis of unknown duration. One other country in WPR recommended treatment of syphilis to be 1.2 MU IM procaine penicillin for 10 days for early syphilis and 1.2 MU IM procaine penicillin plus probenecid 500 mg every 6 h for 15 days for late latent syphilis. Another country in the same region recommended first-line treatment of BPG 0.4MU orally three times per day plus amoxicillin 500 mg orally three times per day. That country guideline also recommended alternative treatments of doxycycline or minocycline100 mg orally every 12 h, or acetylspiramycin 200 mg orally every 3–4 h with both regimens recommended to be used for 2–4 weeks for primary syphilis, 4–8 weeks for secondary syphilis, and 8–12 weeks for “late” syphilis.

#### Treatment and management of exposed infants

Thirteen countries had no specific recommendations on treatment of exposed infants (four from AFR, four from WPR, two from EMR, two from AMR, one from SEAR). Guidelines for 48 (38%) countries recommended treatment with aqueous benzyl penicillin for exposed infants (the WHO first-line recommendation), and 95 (74%) countries recommended procaine penicillin (the WHO alternative recommendation), 99 (77%) countries recommended use of BPG single dose to treat exposed infants. A total of 112 (87%) countries recommended injectable penicillin to treat exposed infants. Ninety-three (82%) countries described recommended follow-up of exposed infants after birth.

#### Comparison of treatment recommendations for exposed infants among high and low prevalence countries

Countries with maternal syphilis prevalence of at least 1% or higher were more likely to recommend aqueous benzyl penicillin to treat exposed infants (55% vs. 29%, *p* = 0.02); these countries were also more likely to mention follow-up with a non-treponemal test at birth for exposed infants (57% vs. 30%, *p* < 0.01) and follow-up examinations at 6–12 weeks (Table 5).

**Table 5.** Treatment for syphilis-exposed infants according to maternal syphilis seroprevalence (n = 115 countries*)

	Maternal syphilis prevalence <1% (n = 73)	Maternal syphilis prevalence ≥1% (n = 42)	*p*-value
Treatment for exposed infant according to WHO^†^ guidelines (penicillin administration)			
Aqueous benzyl penicillin 100,000–150,000 U/kg/day IV for 10–15 days	21 (29%)	23 (55%)	0.02
Procaine penicillin 50,000 U/kg/day single dose IM for 10–15 days	57 (78%)	26 (62)	0.06
BPG 50,000 U/kg/day as single IM dose	59 (81%)	27 (64%)	0.05
Follow-up of exposed infants described			
Infant non-treponemal tests at birth	22 (30%)	24 (57%)	<0.01
Infant non-treponemal tests at 6–12 week	60 (82%)	26 (62%)	0.02
Follow up exam at 6–12 weeks	20 (27%)	23 (56%)	<0.01

*There were 13 countries with no available maternal syphilis seroprevalence data.

BPG = benzathine penicillin G.

^†^World Health Organization.

## Discussion

Syphilis screening for all pregnant women is widely recommended, but not yet in every country. Several countries, including 46 lower-income countries, had no publicly available guidance on syphilis testing or treatment in pregnancy, and an additional 17 countries focused only on syndromic management without discussing screening in pregnancy. Nonetheless, the importance of early screening in pregnancy was reported in over 80% of all guidelines that were identified for this review. Epidemiological and clinical studies support that syphilis screening in the first two trimesters of pregnancy permits infections to be identified and treated early enough to prevent adverse birth outcomes.^14,22^ Unfortunately, countries with higher syphilis prevalence among pregnant women were less likely to specifically recommend early syphilis screening (during the first trimester), suggesting some improvements could be made in future guidance documents.

Nearly half (46%) of the guidelines recommended repeat screening, or suggested repeat screening in the third trimester and/or at delivery regardless of syphilis seroprevalence. WHO does not make specific recommendations for repeated screening in its current guidance documents; however, studies are currently underway evaluating this intervention. Repeat screening can identify new infections in communities with high or rising syphilis prevalence, and has been shown to be a cost-effective public health prevention approach in some settings with higher syphilis prevalence or increasing prevalence in the community.^23,24^

Although injectable penicillin is currently the only drug known to be effective in treating a syphilis-exposed fetus, alternatives to BPG during pregnancy are still commonly used in some countries, especially in the western Pacific (85% of countries), Africa (91%) and South East Asia (100%). Prior WHO guidance documents did not specifically caution against use of alternative treatment strategies, however, the 2016 and 2017 guidelines note that such strategies have not been proven effective in treating an exposed fetus. In particular, oral regimens such as erythromycin and azithromycin must be cautiously recommended to treat syphilis during pregnancy as they are easy to use and thus may be attractive to many countries; however, these regimens may cure the mother while not effectively treating fetal infection or preventing congenital syphilis.^25–27^ Very limited case reports describing use of ceftriaxone during pregnancy exist, and risks to infants have been reported.^28^ WHO is evaluating an alternative treatment regimen for treatment of syphilis when penicillin is unavailable or desensitisation is not feasible.^29^As BPG remains the only antibiotic known to treat an exposed fetus, several countries could benefit by ensuring recommended treatment approaches are the best options to prevent congenital syphilis. Additionally, given the frequent global penicillin shortages,^30^ language that supports forecasting to avoid stock-outs of penicillin and, when stock-outs occur, that prioritises treatment of pregnant women, could decrease potential cases of congenital syphilis.

This review has limitations. Some countries may have guidelines on syphilis screening and treatment during pregnancy and for exposed infants that were not identified because the documents are not widely disseminated or published online. In addition, considering that the recent WHO guidelines on *T. pallidum* were published in 2016 and 2017, some countries may be in the process of revising their guidance which may explain why guidelines were either not identified or, if found, had recommendations inconsistent with the current WHO guidelines. Similarly, screening guidelines for pregnant women may have been limited by a lack of practical testing options. Rapid diagnostic tests for syphilis were only introduced in the years immediately preceding the release of the updated WHO guidelines. National guidelines on syphilis screening in pregnancy may have been influenced by the limited array of available testing modalities, in particular, rapid tests. Another limitation in our analysis comparing guideline recommendations among low and higher maternal seroprevalence countries is that seroprevalence data were not available for 13 countries that had guidelines but had not reported data to the 2015 WHO report.

Syphilis in pregnancy is estimated to occur in approximately one million women per year resulting in greater than 600,000 congenital syphilis cases and 350,000 adverse birth outcomes annually, of which more than 200,000 are stillbirths or neonatal deaths.^2^ Adverse health outcomes, including perinatal death and infant infections, are preventable with early detection and prompt treatment with penicillin of pregnant women, and their sex partners to avoid reinfection. This review indicates that several countries could benefit from developing or updating their guidance on syphilis management during pregnancy according to the newest WHO recommendations. Likewise, some countries could benefit by ensuring that programmes recommending treatment of pregnant women and exposed fetuses and infants, also provide overt guidance on partner management. Updating and adapting guidelines as soon as possible will help address this public health need in countries and also support the global commitment towards the elimination of mother-to-child transmission of syphilis.

## References

[1] Rowley J, Vander Hoorn S, Korenromp E, et al. Chlamydia, gonorrhoea, trichomoniasis and syphilis: global prevalence and incidence estimates, 2016. Bull World Health Organ. 2019;97(8):548–562. doi: 10.2471/BLT.18.228486 31384073 PMC6653813

[2] Korenromp EL, Rowley J, Alonso M, et al. Global burden of maternal and congenital syphilis and associated adverse birth outcomes-Estimates for 2016 and progress since 2012. PloS one. 2019;14(2):e0211720. doi: 10.1371/journal.pone.0211720 30811406 PMC6392238

[3] WHO WHO guidelines for the treatment of Treponema pallidum (syphilis). 2016. [cited 2017 Nov 17]. Available from: http://www.who.int/reproductivehealth/publications/rtis/syphilis-treatment-guidelines/en/27631046

[4] Wijesooriya NS, Rochat RW, Kamb ML, et al. Global burden of maternal and congenital syphilis in 2008 and 2012: a health systems modelling study. Lancet Global Health. 2016;4(8):e525–e533. doi: 10.1016/S2214-109X(16)30135-8 27443780 PMC6759483

[5] Gomez GB, Kamb ML, Newman LM, et al. Untreated maternal syphilis and adverse outcomes of pregnancy: a systematic review and meta-analysis. Bull World Health Organ. 2013;91(3):217–226. doi: 10.2471/BLT.12.107623 23476094 PMC3590617

[6] Blencowe H, Chou VB, Lawn JE, et al. Modelling stillbirth mortality reduction with the lives Saved Tool. BMC Public Health. 2017;17(Suppl 4):784. doi: 10.1186/s12889-017-4742-5 29143647 PMC5688483

[7] WHO Investment case for eliminating mother-to-child transmission of syphilis: promoting better maternal and child health and stronger health systems. 2012. [cited 2016 Oct 28]. Available from: http://apps.who.int/iris/bitstream/10665/75480/1/9789241504348_eng.pdf

[8] WHO Global guidance on criteria and processes for validation: elimination of mother to child transmission of HIV and syphilis. 2017. [cited 2019 Mar 5]. Available from: https://apps.who.int/iris/bitstream/handle/10665/259517/9789241513272-eng.pdf;jsessionid=18DF0496DBC72C60C0A2E04FBE6CF9FE?sequence=1

[9] Warner L, Rochat RW, Fichtner RR, et al. Missed opportunities for congenital syphilis prevention in an urban southeastern hospital. Sex Transm Dis. 2001;28(2):92–98. doi: 10.1097/00007435-200102000-00006 11234792

[10] WHO Guidelines for the management of sexually transmitted infection. 2003. [cited 2018 Jul 5]. Available from: https://www.who.int/hiv/pub/sti/en/STIGuidelines2003.pdf

[11] WHO WHO guidelines for the treatment of Neisseria gonorrhoeae. 2016. [cited 2017 Sep 7]. Available from: http://www.who.int/reproductivehealth/publications/rtis/gonorrhoea-treatment-guidelines/en/27512795

[12] WHO WHO guidelines for the treatment of chlamydia trachomatis. 2016. [cited 2017 Oct 9]. Available from: http://www.who.int/reproductivehealth/publications/rtis/chlamydia-treatment-guidelines/en/27559553

[13] WHO WHO Guidelines for the Treatment of Genital Herpes Simplex Virus. 2016. [cited 2019 Jan 15]. Available from: https://apps.who.int/iris/bitstream/handle/10665/250693/9789241549875-eng.pdf?sequence=11 27875039

[14] WHO Syphilis screening and treatment for pregnant women. 2017. [cited 2017 Dec 9]. Available from: http://apps.who.int/iris/bitstream/10665/259003/1/9789241550093-eng.pdf?ua=1 29757595

[15] Moher D, Liberati A, Tetzlaff J, et al. Preferred reporting items for systematic reviews and meta-analyses: the PRISMA statement. PLoS Med. 2009;6(7):e1000097. doi: 10.1371/journal.pmed.1000097 19621072 PMC2707599

[16] WHO. http://www.who.int/about/regions/en/.

[17] Janier M, Hegyi V, Dupin N, et al. European guideline on the management of syphilis. JEur Acad Dermatol Venereol: JEADV. 2014;28(12):1581–1593. doi: 10.1111/jdv.12734 25348878

[18] SADC Framework for the prevention and control of sexually transmitted infection in the Southern African Development Community (SADC) Region. 2010. [cited 2018 May 17]. Available from: https://www.sadc.int/files/3314/1171/7065/Framework_for_the_Prevention_andControl_of_Sexually_TransmittedInfections_in_the_SADC_Region.pdf

[19] OECS Organization of Eastern Caribbean States (OECS) HIV/STI guidelines. 2017. [cited 2018 Dec 5]. Available from: https://cdn.uc.assets.prezly.com/4ba6d770-7375-4cfe-b08a-8b78f7fdd9e7/-/inline/no/

[20] WHO The use of rapid syphilis test. 2006. [cited 2019 Aug 30]. Available from: https://www.who.int/reproductivehealth/publications/rtis/TDR_SDI_06_1/en/

[21] WHO WHO information note on the use of dual HIV/syphilis rapid diagnostic tests (RDT). 2017. [cited 2019 Aug 30]. Available from: https://apps.who.int/iris/bitstream/handle/10665/252849/WHO-RHR-17.01-eng.pdf?sequence=1

[22] Hawkes SJ, Gomez GB, Broutet N. Early antenatal care: does it make a difference to outcomes of pregnancy associated with syphilis? A systematic review and meta-analysis. PloS one. 2013;8(2):e56713. doi: 10.1371/journal.pone.0056713 23468875 PMC3585307

[23] Taylor MM, Mickey T, Browne K, et al. Opportunities for the prevention of congenital syphilis in Maricopa County, Arizona. Sex Transm Dis. 2008;35(4):341–343. doi: 10.1097/OLQ.0b013e31815bb335 18192931 PMC6785739

[24] Hersh AR, Megli CJ, Caughey AB. Repeat screening for syphilis in the third trimester of pregnancy: a cost-Effectiveness analysis. Obstet Gynecol. 2018;132(3):699–707. doi: 10.1097/AOG.0000000000002795 30095767

[25] Walker GJ. Antibiotics for syphilis diagnosed during pregnancy. Cochrane Database Syst Rev. 2001;3:Cd001143. 10.1002/14651858.CD001143 PMC8407021 11686978

[26] Zhou P, Qian Y, Xu J, et al. Occurrence of congenital syphilis after maternal treatment with azithromycin during pregnancy. Sex Trans Dis. 2007;34(7):472–474. doi: 10.1097/01.olq.0000246314.35047.91 17589329

[27] Kingston M, French P, Higgins S, et al. UK national guidelines on the management of syphilis 2015. Int J STD AIDS. 2016;27(6):421–446. doi: 10.1177/0956462415624059 26721608

[28] Workowski KA, Bolan GA. Sexually transmitted diseases treatment guidelines, 2015. MMWR Recommendations and reports: morbidity and mortality weekly report recommendations and reports. 2015;64(Rr-03):1–137. PMC5885289 26042815

[29] NIH. https://www.clinicaltrials.gov/ct2/show/NCT03752112?term=cefixime%26cond=syphilis%26cntry=BR%26rank=1.

[30] Nurse-Findlay S, Taylor MM, Savage M, et al. Shortages of benzathine penicillin for prevention of mother-to-child transmission of syphilis: an evaluation from multi-country surveys and stakeholder interviews. PLoS Med. 2017;14(12):e1002473. doi: 10.1371/journal.pmed.1002473 29281619 PMC5744908

